# Reply to Supady et al. On the Use of Hemadsorption with CytoSorb in Patients with Septic Shock. Comment on “Kogelmann et al. First Evaluation of a New Dynamic Scoring System Intended to Support Prescription of Adjuvant CytoSorb Hemoadsorption Therapy in Patients with Septic Shock. *J. Clin. Med.* 2021, *10*, 2939”

**DOI:** 10.3390/jcm11051192

**Published:** 2022-02-23

**Authors:** Klaus Kogelmann, Tobias Hübner, Franz Schwameis, Matthias Drüner, Morten Scheller, Dominik Jarczak

**Affiliations:** 1 Klinik für Anästhesiologie und Intensivmedizin, Hans-Susemihl-Krankenhaus GmbH, 26721 Emden, Germany; 2 Department of Anesthesiology and Intensive Care, Kantonsspital Münsterlingen, 8596 Münsterlingen, Switzerland; tobias.huebner@stgag.ch; 3 Department of Anesthesiology and Intensive Care, Landesklinikum Baden-Mödling, 2340 Mödling, Austria; office@schwameis.com; 4 Department of Anesthesiology and Intensive Care Medicine, Klinikum Emden, 26721 Emden, Germany; m.druener@klinikum-emden.de (M.D.); m.scheller@klinikum-emden.de (M.S.); 5 Department of Intensive Care Medicine, University Medical Center Hamburg-Eppendorf, 20251 Hamburg, Germany; d.jarczak@uke.de

Thank you very much for your interest in our research. It is with great attention that we have read your letter to the editor and we thank you for your questions [1], which gives us the opportunity to explain our approach in more detail. We answer your questions and address your concerns point by point.

First, you mentioned that it is unclear how the score was designed and how the parameters were selected. Please see, in Section 2.6 [2], the detailed procedure (“The threshold values are based on Sepsis-3 Criteria…” Perhaps it is not expressed clearly enough that not only the threshold values but also the parameters were selected on this foundation. Among the Sepsis-3 criteria of septic shock, elevated lactate is one of the most important diagnostic criteria. In addition, the norepinephrine requirements are also part of this diagnosis and, according to the Surviving Sepsis Campaign (SSC) guidelines, the demand of volume also plays an important role.

Second, you noted that there is a lack of validation and that we have no comparison with existing scores, such as a Sequential Organ Failure Assessment (SOFA). In the Discussion, we explain that these hemodynamic and sepsis-associated parameters might detect instable patients earlier than score systems, which depend on the worst values in the first 24 h (Discussion Section 4). In summary, in our scoring system, the values chosen belong to the diagnosis criteria of septic shock according to the Sepsis-3 criteria and SSC guidelines. These values are well described and validated. In our work, the severity of the disease in septic shock is represented by the Dynamic Scoring System (DSS) score, that is, the requirements for the development of catecholamine, the use of hydrocortisone and a second catecholamine, as well as volume application and changes in lactate levels. Such dynamic parameters, which are particularly important in septic shock, might represent disease severity more accurately than 24 h scores such as Acute Physiology And Chronic Health Evaluation (APACHE) 2 or Simplified Acute Physiology (SAPS) 2, in which the consideration of such parameters is not included. In the treatment of refractory septic shock, an early initiation of therapy is crucial and, in our opinion, “late“ score systems that reflect the situation of the first 24 h retrospectively are not as useful. Additionally, we want to provide a bedside system to treating physicians that does not depend on several laboratory parameters but rather on clinical signs. The comparison between different DSS scores, and APACHE 2 and SAPS 2 scores are displayed in Table 1.

A comparison between the SOFA and DSS scores would surely be very interesting, so we thank you for this input. Undoubtedly, the SOFA score provides a broader picture of the patients’ clinical status and should definitely not be ignored when deciding to initiate CytoSorb therapy in a septic patient. However, as the primary therapeutic effect of CytoSorb therapy (according to our personal experience and the current published literature) is hemodynamic stabilization and shock control in refractory septic (and vasopolegic) shock states, we want to specifically address this clinical condition and the development in the early hours of treatment with our scoring system, particularly because early changes in this regard might not be reflected as accurately by the SOFA score (due to the longer time necessary for some parameters to react and no difference in SOFA score points for any changes in norepinephrine requirements above 0.1 µg/kg/min).

To correlate the SOFA score of our patients with the mortality rate would have been a very attractive additional analysis; however, it was not possible to perform such analysis at this stage. Nonetheless, this will be considered as a very valuable input for upcoming analyses of our database, which have already started to be discussed internally. As the SOFA score does not reflect changes in norepinephrine requirements beyond 0.1 µg/kg/min, but it does, on the other hand, involve many other important clinical information which is deliberately ignored in our scoring system. There should be certain differences when comparing these two. The pure variety of possible analyses in the current dataset was beyond the scope of the manuscript.

Third, there is no selection criterion for CytoSorb treatment because this research study describes retrospective data. The duration of the therapy is given in Table 2; adsorber change was performed in a mean of 24 h—in fact, this needs to be declared better. The standard care of sepsis was analogous to the SSC guidelines; differences in the centers exist in terms of favoring earlier administration of a second catecholamine vs. volume administration in Münsterlingen vs. the German centers—Hamburg/Emden did not differ. However, particularly in the first 6 h of septic shock treatment, this is also not at all to be expected. The centers provided the following patient numbers: Baden/Austria (*n* = 26), Münsterlingen/Switzerland (*n* = 70), UKE Hamburg (*n* = 124) and Klinikum Emden (*n* = 283). Do-not-resuscitate (DNR) order patients were excluded; this was because they did not receive maximum therapy as treatment was changed by the clinician’s decision after admission to palliative care. Therefore, none of these patients received CytoSorb therapy or maximum standard care. The selected data refer to the period January 2014–January 2020.

As mentioned above, the pure variety of possible analyses of the current dataset would have extended this manuscript infinitely. In addition to Table 1, we compare CytoSorb-treated individuals vs. controls without CytoSorb treatment in Table 1. When comparing these patients, we found differences in baseline characteristics; however, more importantly, patients in the CytoSorb group were more severely ill at T0 and T6, as evidenced by their higher norepinephrine requirements and lactate levels as well as a more frequent administration of hydrocortisone and a second catecholamine. This resulted in significantly higher DSS scores in the CytoSorb group. There was no difference in volume requirements between the groups, suggesting that it was not the “lack” of volume that led to a higher norepinephrine requirement in the CytoSorb group. Despite a more frequent use of a second dose of catecholamine (56% vs. 13.5%) and hydrocortisone (69.1% vs. 30.6%), the norepinephrine dosage was higher in CytoSorb-treated patients (T0, 0.46 vs. 0.3 µg/kg/min; T6, 0.57 vs. 0.43 µg/kg/min).

The multivariate logistic regression model does not translate into a significant treatment benefit of CytoSorb with respect to survival until day 56, and we do not conclude this to be significant. Even if the advantage is not significant, it is a clear signal of a benefit to us. Therefore, we conclude the manuscript as follows:

“The results showed that the use of the CytoSorb device reduced the odds of mortality at day 56 by 44.8%. With regard to the DSS Score, for each one unit increase in score, the odds of mortality at day 56 increased by 23.7% (*p* < 0.001). Similarly, for each additional hour in CytoSorb therapy delay, the odds of mortality at day 56 increased by 1.5% (*p* = 0.034); the associated use of renal replacement therapy (RRT) increased the odds of mortality at day 56 by 75.9%; and lastly, for each one-year increase in patient age, the odds of mortality at day 56 increased by 3.7% (*p* < 0.001)”.

Significances are shown where available.

In summary, in our opinion, there is no lack of information on the choice of parameters—this is clearly explained. A lack of validation is quite normal in a retrospective data analysis and you are right—it should be addressed in a next step, which we also emphasize in the manuscript. The previous study by Schaedler et al. [3] is often used to represent the missing proof of use in a randomized controlled fashion, but any differences in mortality or clinical aspects such as hemodynamic stabilization were not properly (or even not at all) addressed in the study design. The authors reported on the effect of hemoadsorption therapy to reduce cytokines. Furthermore, in that study, treatment was significantly different from current treatments; adsorbers were used for 6 h per day for up to 7 consecutive days, the IL-6 levels were low (around 550 pg/mL) and were measured on day 2, and the authors found a greater reduction at higher IL-6 levels than we did.

Finally, additional data from larger randomized trials would be indeed desirable; however, in our opinion, the DSS score allows one to identify septic patients at an early stage of developing a severe status refractory to standard care in a simple manner, which leads to the use of an adjunctive therapy—i.e., cytokine adsorption: no more but also no less. It is an easy-to-use tool for everyone.

## Figures and Tables

**Table 1:** Baseline characteristics, DSS relevant parameters and outcome variables depending in control patients and Cytosorb-treated individuals.

	Control Group (*n* = 304)	Cytosorb Group (*n* = 198)	*p*-Value
Age (years)	68.59	62.34	<0.0001
APACHE II (points)	37.76	33.87	<0.0001
SAPS II (points)	51.41	59.54	<0.0001
Ventilator days	9.04	13.08	0.003
ICU stay (days)	14.17	19.57	0.002
Hospital stay (days)	22.92	28.60	0.04
ICU mortality (%)	52.36	59.60	0.13
Hospital mortality (%)	57.24	63.41	0.15
Lactate T0 (mmol/L)	4.44	4.76	0.32
Lactate T6 (mmol/L)	4.06	4.73	0.004
Norepinephrine T0 (µg/kg/min)	0.30	0.46	<0.0001
Norepinephrine T6 (µg/kg/min)	0.43	0.57	<0.0002
Second catecholamine T0 (%)	13.49	56.06	<0.0001
Hydrocortisone T0 (%)	30.59	69.19	<0.0001
Volume bolus used (mL/kg)	79.2	78.0	0.56
Dynamic Scoring System (points)	6.64	7.80	<0.0001

**Table 2:** Baseline characteristics, DSS relevant parameters and outcome variables depending on score groups in the CytoSorb group. * *p* < 0.001.

	DSS < 6 (*n* = 17)	DSS 6–8 (*n* = 118)	DSS > 8 (*n* = 63)	*p*-Value (DSS < 6 vs. DSS 6–8)	*p*-Value (DSS < 6 vs. DSS > 8)	*p*-Value (DSS 6–8 vs. DSS > 8)
Age (years)	66.8 (±10.43)	60.5 (±14.80)	64.4 (±15.76)	0.037	0.462	0.097
APACHE II (points)	34.0 (±9.29)	33.4 (±10.23)	34.6 (±10.03)	0.811	0.838	0.473
SAPS II (points)	56.2 (±18.81)	56.6 (±15.30)	65.8 (±19.85)	0.947	0.079	<0.001 *
Ventilator days	7.6 (±8.17)	13.9 (±19.05)	13.0 (±18.53)	0.022	0.247	0.773
ICU stay (days)	12.4 (±8.20)	21.4 (±25.20)	17.9 (±22.85)	0.147	0.123	0.355
Hospital stay (days)	26.1 (±33.70)	30.9 (±37.39)	25.35 (±40.39)	0.599	0.939	0.356
ICU mortality (%)	11 (64.7%)	66 (55.9%)	41 (65.1%)	0.501	0.978	0.235
Hospital mortality (%)	13 (76.5%)	67 (56.8%)	46 (73.0%)	0.122	0.774	0.032
CytoSorb therapy delay (hours)	52.6 (±30.50)	23.0 (±21.50)	18.20 (±20.57)	<0.001 *	<0.001 *	0.138
Number of CytoSorb adsorbers used (n)	2.2 (±0.77)	2.7 (±1.57)	2.7 (±1.58)	0.230	0.117	0.895
Lactate T0 (mmol/L)	3.12 (±3.49)	4.87 (±3.81)	5.01 (±3.26)	0.076	0.041	0.80

## Data Availability

The datasets used and/or analyzed during the current study are available from the corresponding author on reasonable request.
